# Comprehensive Evaluation of Neuropsychiatric and Mucocutaneous Manifestations in the Diagnosis of Systemic Lupus Erythematosus: A Complete Clinical Approach to a Case

**DOI:** 10.7759/cureus.47380

**Published:** 2023-10-20

**Authors:** Héctor Del Río Zanatta, Alexis Zambrano Zambrano, Pablo Belmont Nava, Cristina Lizbeth Puntos Guízar, Julio Martinez Salazar

**Affiliations:** 1 Internal Medicine, National Medical Center November 20, Mexico, MEX

**Keywords:** autoimmune rheumatological conditions, subacute cutaneous lupus erythematosus, mucocutaneous lesions, neuropsychiatric systemic lupus erythematosus (npsle), systemic lupus erythematosus

## Abstract

Systemic lupus erythematosus is a chronic, autoimmune, multisystemic, potentially fatal disease that commonly affects young women between puberty and menopause. It is a multifactorial disease associated with an elevated risk of premature death. The diagnosis is complex due to the broad clinical spectrum as well as the severity at the time of presentation. It is based on clinical manifestations and complementary studies of antibodies. Diagnostic criteria are not available, and classification criteria, such as the ACR/EULAR (American College of Rheumatology/European League Against Rheumatism) of 2019 are often used for diagnosis. Despite its clinical heterogeneity, SLE is an autoimmune disease that can affect multiple systems, and its early diagnosis is essential to avoid damage to vital organs and improve clinical outcomes. This case report shows atypical manifestations of a patient with neuropsychiatric and dermatological symptoms that were essential within the clinical picture to make the diagnosis of systemic lupus erythematosus.

## Introduction

Systemic lupus erythematosus is a chronic, autoimmune, multisystemic, potentially fatal disease that commonly affects young women between puberty and menopause [1]. It has a heterogeneous clinical picture resulting from a cascade of autoimmunity. SLE is a multifactorial disease associated with an elevated risk of premature death so the delay in diagnosis and initiation of treatment increases the risk of damage to vital organs [2]. The diagnosis of SLE is complex due to the broad clinical spectrum as well as the severity at the time of presentation. It is based on clinical manifestations and complementary studies of antibodies. Currently, despite the great advances in the diagnosis and treatment of patients with SLE, this remains a diagnostic challenge due to its clinical variability, the severity of its presentation, and the diagnostic delay with an impact on patient mortality [1-4]. Below, we describe a case that appears to be a diagnostic challenge by presenting unusual clinical manifestations of the disease.

## Case presentation

**Figure 1:**
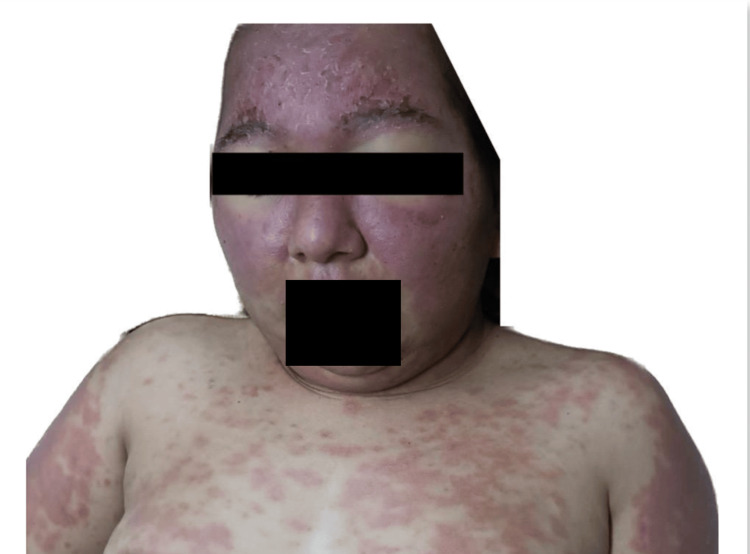
Dermatosis disseminated to the chest, forehead, and malar region

**Figure 2:**
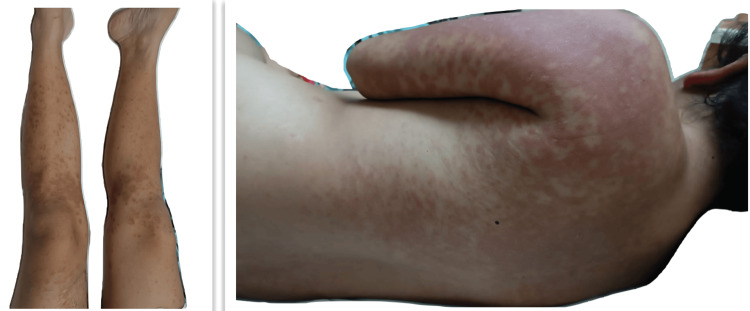
Dermatosis disseminated to the trunk and lower extremities

**Figure 3:**
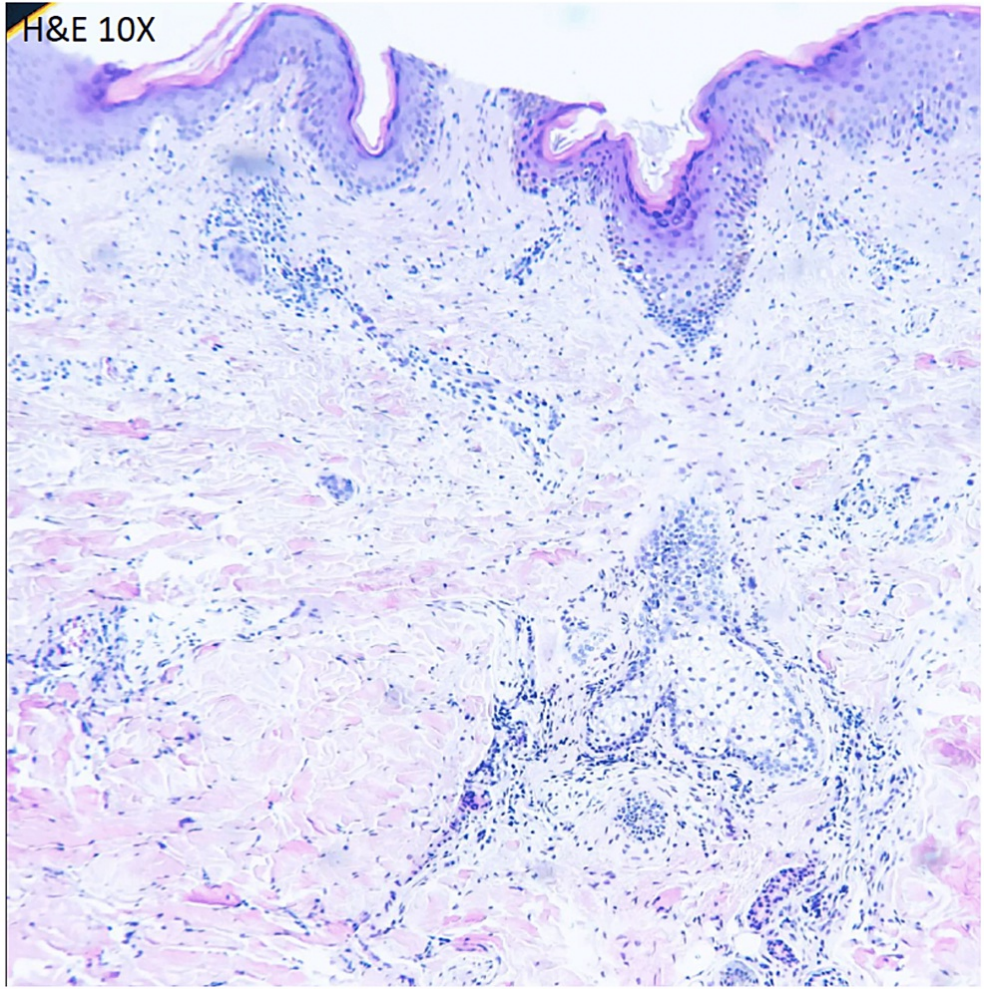
Histopathology test Microscopic description: In histological sections of the skin, the epidermis shows vacuolization of the basal layer, focal apoptosis of keratinocytes, and focal hypogranulosis, and in the superficial dermis, there is a lymphocytic infiltrate with perivascular localization. Mild accumulations of mucin are identified in the superficial dermis. Histochemistry = Alcian blue: positive. Diagnosis: cutaneous lupus erythematosus.

This was a 31-year-old woman with no chronic degenerative history. She began to experience symptoms, including vomiting, diarrhea, and a high fever of 39°C. After a month, she had to be hospitalized due to seizures with the presumed diagnosis of viral encephalitis receiving treatment with intravenous acyclovir without showing improvement. Three months later, she developed dermatosis diffused to the face, arms, the V of the neckline, and the back, with polycyclic lesions characterized by erythema, purpuric macules that alternate with some postinflammatory macules of irregular shape ranging from 2 to 4 cm in diameter that some converge forming larger lesions. There were some pustules and excoriate papules, 0.5 cm in diameter with well-defined edges, on the face and chest (Figures 1, 2). A skin biopsy was performed with lymphocytic infiltrate with perivascular localization (Figure 3).

After documenting a new event of generalized clonic tonic crisis followed by intense stabbing headache, dizziness, chills, and auditory hallucinations, immunological tests were carried out with positive antinuclear antibodies dilution 1:80 with specificity for double-chain DNA (995), and anti-RNP (67.9) (the rest of laboratories are shown in Table 1) meeting ACR/EULAR (American College of Rheumatology/European League Against Rheumatism) classification criteria of 2019 and integrating the diagnosis of systemic lupus erythematosus with neuropsychiatric, and mucocutaneous manifestations. Finally, it was decided to admit the patient to the intensive care unit to be treated with glucocorticoids, gamma globulin, and immunosuppressive agents. 

**Table 1:** Laboratories ANA: Anti-nuclear antibodies; Anti-RNP: Antibodies to ribonucleoprotein; Anti-SM: Anti Smith antibody; Anti-dsDNA: Anti-double stranded DNA; Anti-SSA: Anti-Sjögren's-syndrome-related antigen A autoantibodies; Anti-SSB: Anti-Sjögren's syndrome type B

Test	Result	Reference range
Leukocytes	7.87 /mm3	4.5 - 11 /mm3
Hemoglobin	10.1 g/dL	14 - 18 g/dL
Hematocrit	27.3%	40 - 54 %
Medium corpuscular volume	86.1 fT	84 - 100 fT
Mean corpuscular hemoglobin	31.9 pg	26 - 32 pg
Reticulocytes	2.36	0.5 - 1.5
Platelets	499 /mm3	150 - 500 /mm3
Neutrophils	4.33 /mm3	1.4 - 8 /mm3
Lymphocytes	1.6 /mm3	0.9 - 5.2 /mm3
Complement C3	38.21 mg/dL	90 -180 mg/dL
Complement C4	2.88 mg/dL	10 - 40 mg/dL
Immunologic tests		
ANA	+++	
Nucleolar	++	
Smooth muscle	++	
Anti-RNP	67.9	< 20 negative
AntiSM	16.4	< 20 negative
Anti-dsDNA	995.19 UI/mL	0 - 200 negative
Anti-cardiolipin G	15.71 GPL	<15 negative
Anti-cardiolipin M	7.3 MPL	<12.5 negative
Anti-SSA	70.31	< 20 negative
Anti-SSB	8.75	< 20 negative
Rheumatoid factor	126.42 UI/mL	0 - 15.9 UI/mL
Citrulline C peptide	4.98 U	< 20 negative
Beta 2 glycoprotein IgG	7.27	0.109 - 0.253 mg/dL
Beta 2 glycoprotein IgM	10.96	

## Discussion

SLE is an autoimmune disease characterized by the adhesion of autoantibodies and immune complexes that cause cell, tissue, and organ damage. In most patients, there are autoantibodies years before presenting the clinical symptoms of the disease. More than 90% of the cases correspond to women of reproductive age at the time of diagnosis. The prevalence in Mexico according to the Mexican Registry of Lupus from Universidad Nacional Autónoma de México (UNAM), estimates 20 per 100,000 people. According to race and gender, the highest prevalence is observed in African American and Afro-Caribbean women and the lowest prevalence in Caucasian males [1,2].

The diagnosis is based on the presence of characteristic clinical manifestations and autoantibodies indicative of immune reactivity or inflammation in various organs. Constitutional symptoms, such as weight loss, fatigue, and low-grade fever, are common and may be accompanied by arthralgias or arthritis. Cutaneous manifestations occur in up to 80% of patients as acute, sub-acute, and chronic lesions. SLE should be considered when patients, particularly women of reproductive age, present with hematologic findings, such as thrombocytopenia, leukopenia, lymphopenia, or anemia; renal findings, such as hematuria, proteinuria, cellular casts, or elevated serum creatinine level; respiratory symptoms, such as cough, dyspnea, hemoptysis, or pleuritic pain; or central nervous system (CNS) signs such as headache, photophobia, or focal neurologic deficits [2].

To date, there are no diagnostic criteria available, so classification criteria are used for that purpose. The ACR/EULAR criteria (Table 2) from 2019 have a better combination of sensitivity and specificity at 96% and 93.4%, respectively, requiring positive antinuclear antibodies to titles greater than or equal to 1:80 as an entry criterion, in addition to one or more clinical criterion and 10 or more points. However, it should be noted that fulfilling the classification criteria is not necessary to make a clinical diagnosis of SLE in certain situations [1,2,4].

**Table 2:** European League Against Rheumatism/American College of Rheumatology classification criteria for SLE Adapted from reference [2] ANA: Antinuclear antibody; B2GP1: B2 glycoprotein 1; dsDNA: Double-stranded DNA; HEp-2: Human epithelial type 2; SLE: Systemic lupus erythematosus *In an assay with 90% specificity against relevant disease controls.

Entry criterion: positive ANA test result	
ANA at a titer ≥ 1:80 on HEp-2 cells, or an equivalent positive test result (ever)	
If absent, do not classify as SLE. If present, apply additive criteria	
Additive criteria	
Do not count a criterion if there is a more likely explanation than SLE. The occurrence of a criterion on ≥1 occasion is sufficient	
SLE classification requires ≥1 clinical criterion and ≥10 points	
Criteria need not occur simultaneously	
Within each domain, only the highest weighted criterion is counted toward the total score	
Criteria	Weight
Clinical domains	
Constitutional	
Fever	2
Hematologic	
Leukopenia	3
Thrombocytopenia	4
Autoimmune hemolysis	4
Neuropsychiatric	
Delirium	2
Psychosis	3
Seizure	5
Mucocutaneous	
Nonscarring alopecia	2
Oral ulcers	2
Subacute cutaneous or discoid lupus	4
Acute cutaneous lupus	6
Serosal	
Pleural or pericardial effusion	5
Acute pericarditis	6
Musculoskeletal	
Joint involvement	6
Renal	
Proteinuria >0.5 g per 24 h	4
Renal biopsy class II or V lupus nephritis	8
Renal biopsy class III or IV lupus nephritis	10
Immunologic domains	
Antiphospholipid antibodies	
Anticardiolipin antibodies, anti-B2GP1 antibodies, or lupus anticoagulant	2
Complement proteins	
Low c3 or low c4 level	3
Low c3 and low c4 level	4
SLE specific antibodies	
Anti-dsDNA antibody* or anti-Smith antibody	6
Classify as SLE with a score of ≥10 if the entry criterion is fulfilled	

The estimation of the incidence and prevalence of neuropsychiatric and neurological conditions vary greatly due to the heterogeneity of the definitions and methodology of the studies. Most do not clearly state whether the presentation of neurological symptoms is associated with SLE activity. The prevalence of neuropsychiatric systemic lupus erythematosus (NPSLE) of 21-95% has been described according to the modification of the ACR nomenclature for neurolupus. Our patient within the 19 established clinical manifestations, presented 2 episodes of epileptic seizures (reported in 7-20% within the first five years of onset), psychosis (2-11%), cognitive dysfunction (3-5%), and migraine headache. Risk factors for the development of neuropsychiatric manifestations like a history of psychosis, neuropathy, proteinuria, antiphospholipid antibodies (mainly anticardiolipin), anti-Sm antibodies, hypocomplementemia in c3, and the use of glucocorticoid at variable doses have been described [1,2,4].

Antibodies associated with NPSLE are antiphospholipid antibodies (40%) described for focal manifestations such as transverse myelitis and cerebrovascular disease. Anti-neuronal (60%) was described for diffuse manifestations and anti-ribosomal (10-40%) for psychiatric manifestations (especially psychosis). Anti-NMDA (25-50%) is associated with cognitive dysfunction, anti-aquaporin (4; 2%) with optic neuromyelitis, transverse myelitis, and Sjogren's syndrome [1,2,4].

The dermatological manifestations of our patient are compatible with subacute cutaneous lupus, with a widespread course, a history of photosensitivity without leaving a scar, maculopapular presentation, with small plaques that can have psoriasiform lesions and even flat lichen, in addition to palmoplantar purpuric lesions of non-painful vascular characteristics. The presence of positive anti-RO antibodies has been described in the literature in up to 90% of patients with cutaneous lesions, accompanied by an increase in hair loss, with telogen effluvium of multifactorial cause [5].

The treatment of systemic lupus erythematosus is complex, and at the moment, there have been no major changes in recent years in terms of the recommendations of the EULAR. The objective of the treatment is to achieve the state of low activity of the disease assessed by the scales presented in the guidelines. As for pharmacological management, disease-modifying drugs are still the first line of treatment for SLE. This group includes hydroxychloroquine, cyclosporine, and mofetil mycophenolate. Advances in the development of biotechnological drugs have allowed a significant change in the treatment of these patients. Within the group of biological drugs approved for the treatment of SLE are rituximab, belimumab, anifrolumab, and voclosporin, all with different results in the control of the disease. Some drugs for the treatment of SLE are currently being studied and their efficacy and safety have yet to be reviewed [3].

## Conclusions

The case presented illustrates a highly complex and atypical clinical picture of the challenges in the diagnosis of systemic lupus erythematosus (SLE). It is an autoimmune disease that can affect multiple systems, and its early diagnosis is essential to avoid damage to vital organs and improve clinical outcomes. The patient experienced unusual manifestations, including neuropsychiatric and dermatological symptoms, which initially led to an uncertain diagnosis. The presence of high fever, auditory hallucinations, sharp headaches, polymorphic skin lesions, and episodes of epileptic seizures added complexity to the clinical landscape. The detection of autoimmune markers, such as ANA, anti-double-stranded DNA, and anti-RNP antibodies, along with clinical suspicion and hypocomplementemia finally led to the diagnosis of SLE after meeting the 2019 ACR/EULAR classification criteria with neuropsychiatric and mucocutaneous manifestations. This case emphasizes the importance of a comprehensive evaluation of patients with unusual or atypical symptoms and highlights the need for a multidisciplinary approach to properly address this type of complex clinical condition. Ultimately, the case emphasizes the importance of promoting early diagnosis and adequate treatment to improve the quality of life of patients and reduce the risk of serious complications.
